# The Role of Inflammation in the Treatment of Schizophrenia

**DOI:** 10.3389/fpsyt.2020.00160

**Published:** 2020-03-18

**Authors:** Guillaume Fond, Christophe Lançon, Theo Korchia, Pascal Auquier, Laurent Boyer

**Affiliations:** Hôpitaux Universitaires de Marseille (HUM), Aix-Marseille University, School of Medicine - La Timone Medical Campus, EA 3279: CEReSS - Health Service Research and Quality of Life Center, Marseille, France

**Keywords:** inflammation, schizophrenia, treatment, anti-inflammatory, cytokines

## Abstract

**Background:** Inflammation plays a major role in the onset and maintenance of schizophrenia. The objective of the present work was to synthetize in a narrative review the recent findings in the field of inflammation in schizophrenia and their application in daily practice.

**Method:** This review was based on the most recent meta-analyses and randomized controlled trials.

**Results:** The disturbed cytokines depend on the phase of the illness. A meta-analysis of cytokines in schizophrenia found higher levels of pro-inflammatory and anti-inflammatory cytokines in the peripheral blood in both patients with first-episode schizophrenia and relapsed patients than in healthy controls. Exploring detailed data on immune-inflammatory disturbances in SZ reveals that IL-6 is one of the most consistently disturbed cytokines. Other cytokines, including IL1, TNF, and IFN, are also disturbed in schizophrenia. Choosing a broad spectrum anti-inflammatory agent that may inhibit subsequent pathways might be particularly useful for the treatment of inflammatory schizophrenia. Highly sensitive C-Reactive Protein is a useful screening marker for detecting inflammation in SZ subjects. Anti-inflammatory agents have shown effectiveness in recently published meta-analyses. Only one study found a significant difference between celecoxib and placebo, but two found a trend toward significance on illness severity and one on positive symptoms. In addition, other published and unpublished data were included in another meta-analysis that concluded the significant effect of add-on celecoxib in positive symptoms in first episode patients. There is a lack of data to determine if aspirin is truly effective in schizophrenia to date. Other anti-inflammatory agents have been explored, including hormonal therapies, antioxidants, omega 3 fatty acids, and minocycline, showing significant effects for reducing total, positive, and negative score symptoms and general functioning. However, each of these agents has multiple properties beyond inflammation and it remains unclear how these drugs improve schizophrenia.

**Conclusion:** The next step is to tailor anti-inflammatory therapy in schizophrenia, with two main challenges: 1. To provide a more efficient anti-inflammatory therapeutic approach that targets specific pathways associated with the pathology of schizophrenia. 2. To develop a more personalized approach in targeting patients who have the best chance of successful treatment.

## Introduction

Though conventional treatments have improved schizophrenia prognosis, they and the response rate of antipsychotics in schizophrenia remain unsatisfactory. The antipsychotics introduced in the 1950s have shown moderate global effectiveness with a mean effect size of 0.38 (1). The response rate of clozapine—the most effective antipsychotic—is only 33% after 3 months of treatment (1). Antipsychotics are effective on positive symptoms but unsatisfactory on negative/depressive symptoms, social functioning, and quality of life (1).

An explanation for this high rate of non-response and relapses relies on the observation that current pharmacological treatments are primarily based on the monoaminergic hypothesis, without involving the personalized medicine approach. According to this hypothesis, schizophrenia is principally due to a dopamine dysfunction in the brain (with an excess in the striatum ventral tegmental area and a deficit in the prefrontal cortex). All current antipsychotics target dopamine deficits in the brain. Yet clozapine, the antipsychotic that has shown the best effectiveness, has also one of the lowest potentials to reduce dopamine in the brain (2). This paradox remains unsolved to date.

The high rate of therapeutic failure in psychiatry can most likely be accounted for by the limitations pertaining to brain-orientated treatments. Current treatments do improve neurotransmitter deficits, but without addressing the source of these deficits. This may explain the high relapsing rates and chronic illness causes.

The objective of the present review was to synthetize the state of knowledge of the role of inflammation in the treatment of schizophrenia.

## Materials and Methods

This review was based on the most recent meta-analyses and randomized controlled trials, and if these were not available, on preliminary data. The Medline® database was explored from its inception to September 10th 2019, without language restriction. The research paradigm was: (schizophrenia) AND (inflammation OR anti-inflammatory agents OR cytokines OR C reactive-protein). The references of each article were also checked. Given the broad spectrum of the subject, the systematic review design was not adapted to the present work and a narrative review form has been preferred. Thus, no flow-chart or study quality assessment has been provided.

## Results

Two thousand two hundred seventy-eight articles were identified in the Medline search. Of them, 41 were included in the present review.

### The Role of Inflammation in the Pathogenesis and Maintenance of Schizophrenia

The pathophysiological underpinnings of inflammation and its potential role in schizophrenia onset and maintenance have been previously synthetized (3). Schizophrenia is characterized by risk genes that promote inflammation, and by environmental stress factors and alterations of the immune system. Neuromediator alterations classically described in schizophrenia (dopamine, serotonine, glutamate) have also been identified in low-level neuro inflammation and may be key triggers of schizophrenia symptoms onset and maintenance (4). The contribution of chronic inflammation to major mental disorders has received increased attention, revealing a host of pharmacologic targets. Indeed, multiple recent reviews clearly demonstrate that schizophrenia is associated with a dysregulation of immune responses, as reflected by the observed abnormal profiles of circulating pro- and anti-inflammatory cytokines in affected patients (5). Impaired central nervous system volume and microglial activations in schizophrenia have been confirmed in neuroimaging studies (4).

Among potential sources of chronic low-grade inflammation, infectious agents and environmental toxins (including tobacco smoke and cannabis) have been identified (6–11). It may also be a secondary reaction to trauma-related neuronal lesions or a genetic effect (4).

Microglia constitute between 10 and 20% of all cells in the CNS and are the most important component of the local CNS immune system (12). Microglia is activated in case of injury or disease such as systemic infection, and is involved in the activation of cytokines, the key mediators of inflammation.

A variety of low-level stimuli including aging, neurodegeneration and stress can also cause microglia to be “sensitized” or “primed,” a process that elicits an exaggerated immune response (4). Once microglia are primed, an additional low-level stimulus, e.g., minor systemic inflammation, may exacerbate or re-exacerbate an immune response in the CNS with behavioral consequences (13).

### Inflammatory Markers in Schizophrenia

Fibrin is a protein that is increased in inflammatory processes. Degradation products of fibrin have been found in postmortem brains of schizophrenia patients and in the cerebrospinal fluid (CSF) of about 50% of them (14). The density of microglia is significantly increased in schizophrenia [mostly in the temporal cortex (14)] yet with substantial heterogeneity between studies. Astrocytes and oligodendrocytes' densities did not differ significantly between schizophrenia and healthy controls. The results of postmortem histology are paralleled with an overall increase in expression of proinflammatory genes in SZ patients, while anti-inflammatory gene expression levels were not different between SZ patients and controls. These results strengthen the hypothesis that immune system disturbances are involved in the pathogenesis of schizophrenia.

A meta-analysis of cytokines in schizophrenia found higher levels of proinflammatory cytokines in the peripheral blood in both patients with first-episode schizophrenia and relapsed patients than in healthy controls, but it also found higher levels of some anti-inflammatory cytokines in these patients than in controls (15). The disturbed cytokines depend on the phase of the illness.

In first-episode psychosis, interferon-γ (IFN-γ), IL-1RA, IL-1β, IL-6, IL-8, IL-10, IL-12, sIL-2R, TGF-β, and TNF were all significantly increased, and levels of IL-4 were significantly decreased. Age, sex, illness duration, smoking, and BMI were all unrelated to IL-6 and TNF-α increase in first-episode psychosis.

In acute exacerbation of chronic SZ, an increase of IFN-γ, IL-1RA, IL-1β, IL-6, IL-8, IL-12, sIL-2R, TGF-β, and TNF alongside a decrease of IL-4 and IL-10 levels were found in SZ compared to controls.

In chronically ill SZ, IL-6, TNF, sIL-2R, IL-1β were increased and IFN-γ was decreased in SZ patients compared to controls, with no significant difference in the levels of IL-2, IL-4, or IL-10. Age, sex, illness duration, smoking, and BMI were all unrelated to the association between IL-6 and SZ. Of note, a meta-analysis of cytokines in the CSF of SZ showed increased levels of IL-6 and IL-8 (16).

Inflammation has been bilaterally associated with cortisol disturbances (17). Cortisol disturbances have shown associations with treatment non-response in schizophrenia and major depression, which is frequent in SZ patients (18).

In summary, IL-6 is the most consistent increased cytokine in all phases of schizophrenia, but a large bundle of other cytokines is found to be disturbed. These findings suggest that choosing a broad-spectrum anti-inflammatory agent that may inhibit subsequent pathways may be particularly useful for the treatment of inflammatory schizophrenia.

### Anti-inflammatory Therapies Tested So Far in Schizophrenia

A detailed overview of the efficacy of anti-inflammatory treatment in schizophrenia was published in 2014 and provides one of the most convincing pieces of evidence that inflammation is involved in schizophrenia (19). This work has been recently updated (20). Sixty-two double-blind randomized clinical trials including 2,914 SZ patients were included in the latter.

The cyclooxygenase (COX) inhibitors were the first anti-inflammatory agents to be tested in schizophrenia in the early 2000's. The prostaglandin inflammatory cascade is activated by two COX enzymes named COX-1 and COX-2. The COX 1 is a permanent/state COX responsible for the baseline inflammatory response (e.g., reacting to a wound). The COX-2 is activated only in case of acute inflammation (in case of infection for example) (21).

That's why celecoxib, a specific COX-2 inhibitor, has been the first and most studied COX-targeted anti-inflammatory agent in schizophrenia. Four RCTs investigated the effects of celecoxib in 195 patients (22–24) with inconsistent findings. Only one study found a significant difference between celecoxib and placebo (24), but two found a trend toward significance on PANSS total score (*p* = 0.06 for both) and one on PANSS positive score (*p* = 0.05) (22). In addition, other published and unpublished data were included in another meta-analysis that concluded the significant effect of add-on celecoxib in SZ in PANSS total and PANSS positive scores in first episode SZ patients (25).

COX-1 inhibitor (low-dose aspirin) has been studied in two RCTs, with positive results on all PANSS scores in one study (26), and a positive but small effect on PANSS total- and positive score in the other (27). Aspirin is to date the anti-inflammatory agent that has shown the greatest potential for effectiveness in schizophrenia (20). This effect was driven by a high-baseline PANSS score subgroup. Yet the methodology of these trials has been questioned, especially due to the differences in antipsychotic treatments in each groups and the statistically significant but clinically non-significant effect reported in these trials (28). In summary, there is a lack of data to determine if aspirin is truly effective in SZ to date. Moreover, aspirin is at increased risk of ulcer and hemorrhagic side effects, limiting its prescription.

Other anti-inflammatory agents have been explored, yet with a broad spectrum of other properties. These agents included hormonal therapies, antioxidants, omega 3 fatty acids, and minocycline, an antibiotic that penetrates the brain. Overall, anti-inflammatory agents (mostly celecoxib, aspirin, minocycline) have shown significant effects for reducing total, (effect size = 0.41, 95% confidence interval (CI) = [0.26, 0.56]), positive (effect size = 0.31, 95% CI = [0.14, 0.48]), and negative (effect size = 0.38, 95% CI = [0.23, 0.52]) scores in the PANSS. General functioning was also significantly enhanced by overall anti-inflammatory agents. However, each of these agents has multiple properties beyond inflammation (e.g., hormonal for estrogens/pregnelonone, antibiotic/glutamatergic for minocycline, antioxidant for N-acetyl-cysteine) and it remains unclear how these drugs improve schizophrenia.

## Discussion/Perspectives

### Schizophrenia Patients With Chronic Low-Grade Peripheral Inflammation: The Best Candidates for Anti-inflammatory Treatment

To improve anti-inflammatory drug effectiveness, it is necessary to identify best candidate SZ patients using inflammatory markers. This is contrary to previous studies, which only included SZ patients using clinical criteria [for review see (21)]. This has led to high heterogeneity in previous meta-analyses (25). We have seen that defining an inflammation signature in schizophrenia was difficult due to the multiple cytokines that may be disturbed according to the state of the illness. We have recently published a review on the interest of hs-CRP to identify peripheral inflammation in schizophrenia (29). Hs-CRP is the most common peripheral marker of inflammation and is synthesized by the liver in response to IL-1 and IL-6 according to the following pathway. It has been reliably used in multiple randomized controlled trials for exploring the role of inflammation in treatment response (30–34). Recent data indicate that blood CRP concentrations have been associated with high central glutamate, which correlated with symptoms of anhedonia, one of the symptoms of schizophrenia (35).

In stabilized SZ patients, around one third exhibit high CRP levels (>3 mg/L) (36). These patients were found to have more resistance to conventional treatments and more cognitive impairment, which confirms the clinical interest of targeting this specific subgroup of patients (36, 37).

The blood–brain barrier protects the brain from peripheral inflammation, and the cytokines state in the blood does not reflect the situation in the brain. Yet different pathways exist between the peripheral and the CNS immune systems. Hs-CRP appears to be a good reflector of central inflammation in non-SZ populations (35). It seems also well-suited for guiding immunotherapies targeting IL-6 (35).

In summary, hs-CRP is a useful screening marker for detecting inflammation in SZ subjects.

### Janus-Kinase Inhibitors (JAKinibs): A Promising Treatment for Inflammatory Schizophrenia

We have seen that schizophrenia was associated with a broad range of disturbed cytokines. These cytokines bind to receptors that activate downstream the so-called JAK/STAT signaling pathway (38) involved in gliogenesis, synaptic plasticity, microglia activation and neurogenesis, all implicated in the pathophysiology of schizophrenia (39). Moreover, depressive symptoms are frequent in schizophrenia and the antidepressant actions of current treatments have been confirmed to be mediated by JAK/STAT-dependent mechanisms (40). Small-molecule inhibitors of JAKs (jakinibs) have been shown as safe and efficacious options for the treatment of rheumatoid arthritis, psoriasis, and inflammatory bowel disease (41), and may be promising treatments for schizophrenia that should be evaluated.

### To Destroy the Root Cause of the Evil: Addressing the Sources of Inflammation

Adding an anti-inflammatory agent may be not sufficient if the potential sources of inflammation are not addressed. Among them, tobacco smoking, Toxoplasma latent infection, microbiota disturbances, lack of physical activity, and poor diet have been identified as major modifiable sources of inflammation in SZ patients that should be addressed in schizophrenia daily care (7–10). Tobacco smoking cessation, Mediterranean or anti-inflammatory diets, and physical activity appear as promising interventions to be tested in inflammatory SZ patients, yet further studies are needed to determine their effectiveness.

## Limits

This review has shown one major limit in the field of inflammation in schizophrenia, i.e., the definition of a consensual inflammatory signature to determine which patients may benefit from anti-inflammatory strategies. While TSPO-PET imaging appears as the gold standard to explore neuro-inflammation to date (42), its costs and its dissemination (limited by MRI availability and genotyping) prevents it from being widely distributed. Hs-CRP appears as a potentially good biomarker, but further studies should confirm if peripheral CRP is a good marker of central neuro-inflammation in schizophrenia, as suggested in one study in depression (35).

## Conclusion

The next step is to tailor anti-inflammatory therapy with the best response and highest safety in schizophrenia. There are two main challenges:

- to provide a more efficient anti-inflammatory therapeutic approach that targets specific pathways associated with the pathology of schizophrenia. Exploring detailed data on immune-inflammatory disturbances in schizophrenia reveals that IL-6 is one of the most consistently disturbed cytokines in SZ. Other cytokines including IL1, TNF, and IFN are also disturbed in schizophrenia.
- to develop a more personalized approach in targeting patients who have the best chance of successful treatment. We hypothesize that SZ patients with chronic low-grade peripheral inflammation (SZ-CPI) defined by hs-CRP blood level ≥3 mg/L (a reliable marker used in previous works) make the best candidates for anti-inflammatory treatments.

## Author Contributions

GF and LB wrote the first draft of this work. All authors reviewed and validated the final version of the manuscript.

### Conflict of Interest

The authors declare that the research was conducted in the absence of any commercial or financial relationships that could be construed as a potential conflict of interest.
